# Nanoparticle-Mediated Drug Delivery of Doxorubicin Induces a Differentiated Clonogenic Inactivation in 3D Tumor Spheroids In Vitro

**DOI:** 10.3390/ijms24032198

**Published:** 2023-01-22

**Authors:** Roxana Cristina Popescu, Verena Kopatz, Ecaterina Andronescu, Diana Iulia Savu, Wolfgang Doerr

**Affiliations:** 1 Department of Life and Environmental Physics, National Institute for R&D in Physics and Nuclear Engineering “Horia Hulubei”, Reactorului Street, 30, 077125 Magurele, Romania; 2 Department of Science and Engineering of Oxide Materials and Nanomaterials, Politehnica University of Bucharest, Gheorghe Polizu Street, 1-7, 011061 Bucharest, Romania; 3 Department of Bioengineering and Biotechnology, Politehnica University of Bucharest, Gheorghe Polizu Street, 1-7, 011061 Bucharest, Romania; 4 Department of Radiation Oncology, Medical University of Vienna, Waehringer Guertel, 18-20, 1090 Vienna, Austria

**Keywords:** iron oxide nanoparticles, drug delivery, doxorubicin, radiosensitization, in vitro 3D tumor spheroids

## Abstract

Involvement of 3D tumor cell models in the in vitro biological testing of novel nanotechnology-based strategies for cancer management can provide in-depth information on the real behavior of tumor cells in complex biomimetic architectures. Here, we used polyethylene glycol-encapsulated iron oxide nanoparticles for the controlled delivery of a doxorubicin chemotherapeutic substance (IONP_DOX_), and to enhance cytotoxicity of photon radiation therapy. The biological effects of nanoparticles and 150 kV X-rays were evaluated on both 2D and 3D cell models of normal human keratinocytes (HaCaT) and tumor cells—human cervical adenocarcinoma (HeLa) and human squamous carcinoma (FaDu)—through cell survival. In all 2D cell models, nanoparticles were similarly internalized in a peri-nuclear pattern, but resulted in different survival capabilities following radiation treatment. IONP on normal keratinocytes showed a protective effect, but a cytotoxic effect for cancer cells. In 3D tumor cell models, IONP_DOX_ were able to penetrate the cell spheroids towards the hypoxic areas. However, IONP_DOX_ and 150 kV X-rays led to a dose-modifying factor DMF_SF=0.1_ = 1.09 ± 0.1 (200 µg/mL IONP_DOX_) in HeLa spheroids, but to a radioprotective effect in FaDu spheroids. Results show that the proposed treatment is promising in the management of cervical adenocarcinoma.

## 1. Introduction

Multimodal cancer treatment is a frequent approach for oncologic management, consisting of chemotherapy, which targets the growth inhibition of tumor cells, followed by complete elimination of the malignant tissue, and radiation treatment to remove any modified cells left in the body, as well as adjuvant immunotherapy. Targeted therapies use specific agents, which are directed to act against specific properties of the cancer cells, such as their accelerated metabolism [1,2,3], proteins on the membrane [4,5], associated immune cells [4,6,7], etc.

Nanoparticle-based therapies can be applied in the management of certain cancers in order to directly deliver small quantities of drugs at the tumor site, which concentrates the dose of the active substance at the desired location, while sparing the healthy tissues met in systemic circulation. Here, iron oxide nanoparticles are used, due to their magnetic transport ability [8,9], as well as biocompatibility for normal tissues, proven through their clinical use [10,11,12,13].

Additionally, a combined treatment using radiotherapy and high Z nanoparticles [14,15] has the advantage of improving the final dose response, due to the secondary radiation emitted at the interaction between the applied incident fascicle and the atoms in the nanoparticle. This fact is favored by an enhanced internalization and retention of the radiosensitizing agent (i.e., the nanoparticles) inside the tumor cells [16].

Here, we tested the biological effect of iron oxide-based drug delivery systems of a doxorubicin chemotherapeutic substance in 3D tumor cell models in vitro, as well as a clear cytotoxic differentiated effect between normal and cancer cells. The nanoparticles were previously developed using a modified chemical co-precipitation method [16], which results in stable nano-systems that are able to penetrate the cancer cells, and can be retained for more than one cell cycle, in order to perform as radiosensitizing agents [16,17].

The novelty of this study consists of a proof of concept of dual chemotherapy–radiosensitization efficiency of iron oxide nanoparticles in 3D tumor cells models in vitro. For this, we obtained two spheroid cell models, namely for human cervical adenocarcinoma (HeLa) and human squamous cell carcinoma (FaDu). The efficiency of the technology was proven in comparison to classical 2D cell models, as well as normal keratinocyte cells (HaCaT) using the clonogenic survival assay.

## 2. Results and Discussion

In order to evaluate the radiosensitization potential of IONP drug delivery systems, two-dimensional cell cultures of two cancerous cell lines (FaDu human squamous cell carcinoma and HeLa human cervical adenocarcinoma) and one normal cell line (HaCaT human keratinocytes) were used. The cells were incubated with IONP for 16 h and then irradiated using 150 kV X-rays. This interaction time between the nanoparticles and the cells was lower than one complete cell cycle, and was sufficient to prevent any possible dilution of the internalized nanoparticles due to cell division [16,17].

3D tumor cell cultures were obtained using a liquid-overlay technique [18] (Supplementary Materials, Section S1, Figures S1–S5). In this case, the cells were incubated with IONP for 48 h, then irradiated using different doses of 150 kV X-rays. The interaction time was selected at this time point following the addition of the nanoparticles, because it provided the best IONP penetration effectiveness towards the center of the spheroid, while still preserving its compact morphology (Supplementary Materials, Section S2, Figures S6–S9).

Although superficial radiotherapy using 150 kV X-rays does not provide a high penetration depth in tissues [19], it is suitable for the treatment of surface cancers, such as squamous cell carcinoma and cervical adenocarcinoma. Moreover, it has been shown that in the case of nanoparticle-mediated radiotherapy, the efficiency of the radiosensitization is inversely proportional to the radiation energy [17,20,21,22].

The radiosensitization efficiency of the nanoparticles is not only dependent on the properties of the incident radiation, but also on the biological behavior of the nanoparticles, such as the internalization and penetration abilities of tumors. Thus, in order to evaluate the IONP internalization in both 2D and 3D cell cultures, optical microscopy was employed, using an adequate staining method for the iron-based nanoparticles, with respect to the cells. Moreover, specific areas in the 3D tumor models were stained appropriately to emphasize the metabolically active cells in the synthesis (S) phase of the cell cycle at the exterior area, with respect to the hypoxic cells in the central area of the spheroid.

**Figure 1:**
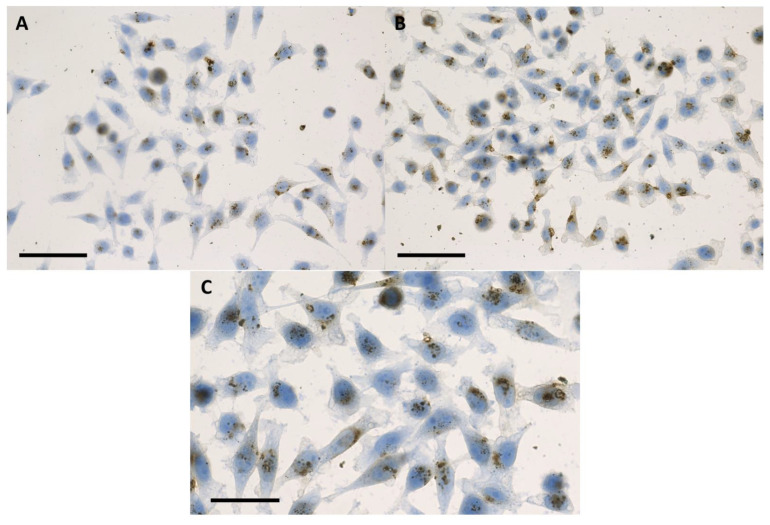
Bright-field microscopy images of human cervical adenocarcinoma (HeLa) cells at 24 h following 16 h of incubation with 100 μg/mL (**A**), and 200 μg/mL (**B**) IONP_DOX_; blue hematoxylin, dark brown IONP; the scale bar is 50 μm; (**C**) detailed image of HeLa cells at 24 h following 16 h of incubation with 200 μg/mL IONP_DOX_; the scale bar is 25 μm.

**Figure 2:**
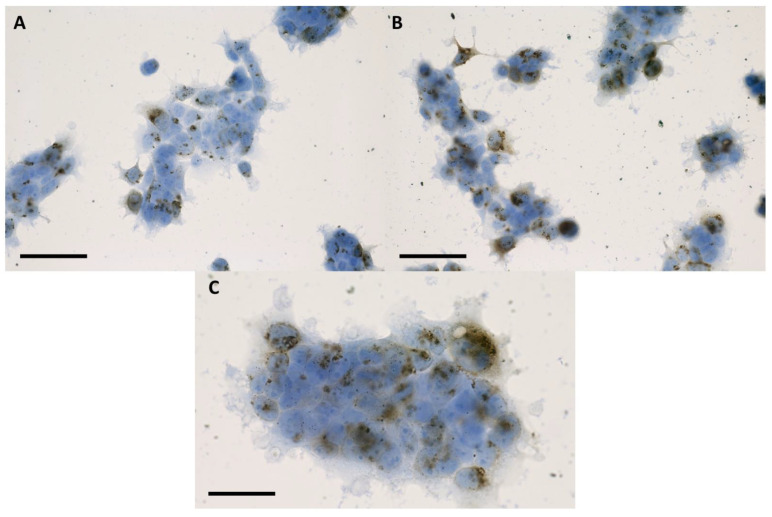
Bright-field microscopy images of human squamous cell carcinoma (FaDu) at 24 h following 16 h of incubation with 100 μg/mL (**A**), and 200 μg/mL (**B**) IONP_DOX_; blue hematoxylin, dark brown IONP; the measure bar is 50 μm; (**C**) detailed image of FaDu cells at 24 h following 16 h of incubation with 200 μg/mL IONP_DOX_; the scale bar is 25 μm.

**Figure 3:**
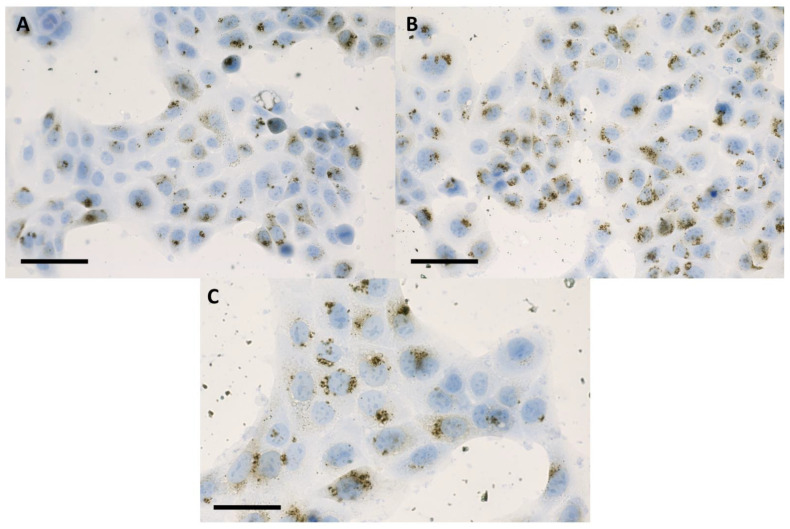
Bright-field microscopy images of human keratinocytes (HaCaT) cells at 24 h following 16 h of incubation with 100 μg/mL (**A**), and 200 μg/mL (**B**) IONP_DOX_; blue hematoxylin, dark brown IONP; the measure bar is 50 μm; (**C**) detailed image of HaCaT cells at 24 h following 16 h of incubation with 200 μg/mL IONP_DOX_; the scale bar is 25 μm.

The discussion concerning a suitable internalization pattern of the nanoparticles for radiosensitization purposes goes beyond the detection of nanoparticles inside the cells. Retention of the nanoparticles following the incubation period must be fulfilled in order to ensure a maximum effect [16,17]. After 16 h of incubation in 2D with nanoparticles, the internalized IONP were not present in the cell nuclei, remaining organized as dark brown-black aggregates that covered the exterior of the nuclear membrane (Figure 1 and Figure 2). The presence of the nanoparticles was evidenced in the cytoplasm of the tumor cells, especially in the peri-nuclear area (Figure 1 and Figure 2). A similar internalizing pattern was also observed in normal cells (Figure 3).

**Figure 4:**
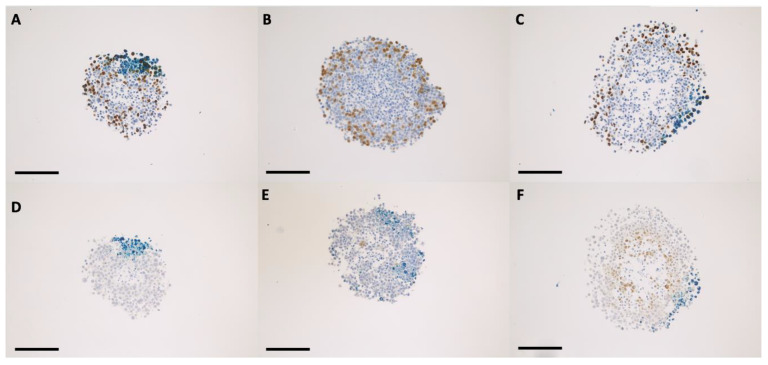
Bright-field microscopy images for HeLa spheroid sections that were previously incubated for 48 h with 200 μg/mL IONP_DOX_; immunohistochemical staining of proliferative areas using bromodeoxyuridine (**A**–**C**), and of hypoxic areas using pimonidazol (**D**–**F**); light blue hematoxylin, dark blue IONP, dark brown proliferative cells, light brown hypoxic cells; measure bar is 100 μm.

**Figure 5:**
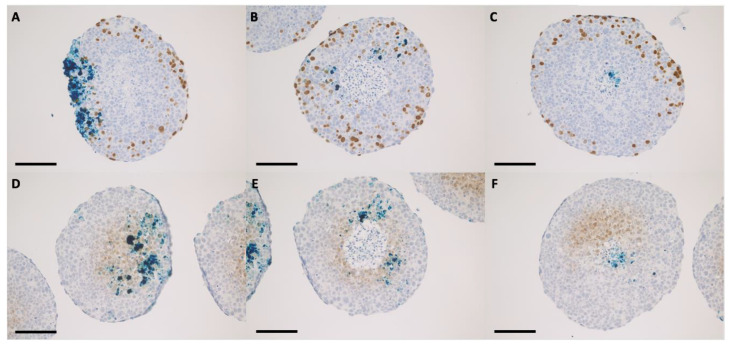
Bright-field microscopy images for FaDu spheroid sections that were previously incubated for 48 h with 200 μg/mL IONP_DOX_; immunohistochemical staining of proliferative areas using bromodeoxyuridine (**A**–**C**), and of hypoxic areas using pimonidazol (**D**–**F**); light blue hematoxylin, dark blue IONP, dark brown proliferative cells, light brown hypoxic cells; measure bar is 100 μm.

**Figure 6:**
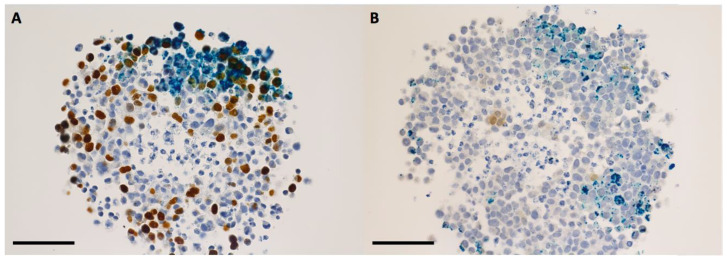
Bright-field microscopy images—details of HeLa spheroid sections that were previously incubated for 48 h with 200 μg/mL IONP_DOX_; immunohistochemical staining of proliferative areas using bromodeoxyuridine (**A**), and of hypoxic areas using pimonidazol (**B**); light blue hematoxylin, dark blue IONP, dark brown proliferative cells, light brown hypoxic cells; measure bar is 50 μm.

**Figure 7:**
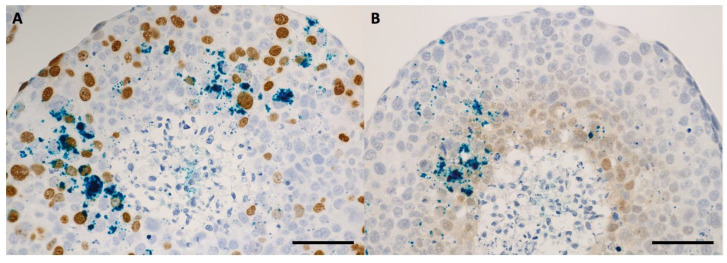
Bright-field microscopy images—details of FaDu spheroid sections that were previously incubated for 48 h with 200 μg/mL IONP_DOX_; immunohistochemical staining of proliferative areas using bromodeoxyuridine (**A**), and of hypoxic areas using pimonidazol (**B**); light blue hematoxylin, dark blue IONP, dark brown proliferative cells, light brown hypoxic cells; measure bar is 50 μm.

**Figure 8:**
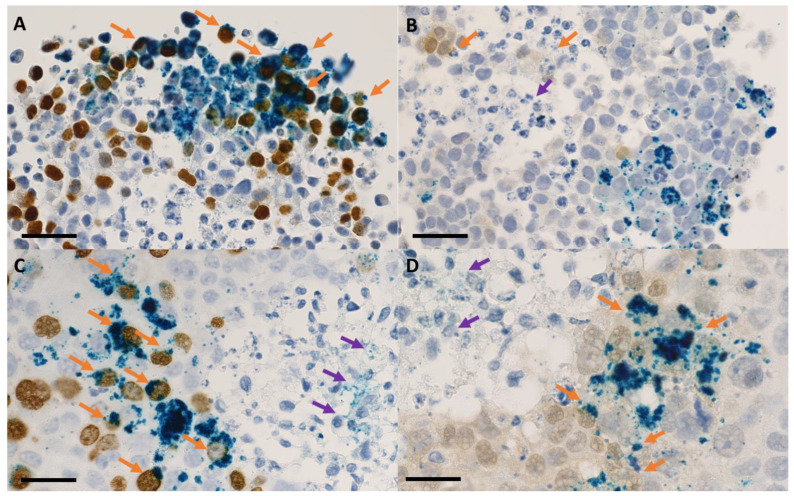
High magnification of bright-field microscopy images—spheroid sections that were previously incubated for 48 h with 200 μg/mL IONP_DOX_: (**A**,**B**) HeLa, (**C,D**) FaDu; immunohistochemical staining of proliferative areas using bromodeoxyuridine (**A**,**C**), of hypoxic areas using pimonidazol (**B**,**D**); light blue hematoxylin, dark blue IONP, dark brown proliferative cells, light brown hypoxic cells; measure bar is 25 μm; orange arrows highlight nanoparticles internalized in proliferative/ hypoxic cells and purple arrows highlight nanoparticles internalized in necrotic cells.

The nanoparticle internalization in the 3D tumor cell models took place according to a concentration gradient, starting from the external upper area of the spheroid body towards its center and through the opposite side, in the sense of the gravitational force (Figure 4 and Figure 5). The maximum degree of nanoparticles penetration through the middle of the spheroids was observed at 48 h of incubation for both tumor cell models (Figure 6, Figure 7 and Figure S6–S9). This incubation time interval was selected for the following radiation experiments. In case of 3D tumor cervical adenocarcinoma, the IONP were localized especially in the proliferative cells area (cells in the synthesis phase of the cell cycle, Figure 4, Figure 6 and Figure 8A,B arrows). On the other hand, in case of the FaDu cell model, the IONP were internalized in both proliferative as well as in the hypoxic areas (Figure 5, Figure 7 and Figure 8C,D arrows).

The long-term response of IONP treatment followed by ionizing radiation exposure was evaluated using the clonogenic assay, in order to reveal possible effects of chemo- and/or radiosensitization upon the cells’ survival ability.

IONP_DOX_ alone induced a cytotoxic effect in all cell models which was mostly independent of concentration, measured through the cells’ survival ability in the absence of ionizing radiation. In the case of 2D-cultured squamous cell carcinoma cells, the intrinsic toxicity of IONP_DOX_ induced a survival of 0.64 ± 0.2 at a concentration of 100 µg/mL, and 0.67 ± 0.06 for 200 µg/mL IONP_DOX_. The survival fraction in case of 2D cervical adenocarcinoma was about 0.78 ± 0.08 at 100 µg/mL, and 0.73 ± 0.04 at 200 µg/mL for DOX-loaded IONP. The nanosystems’ cytotoxic effects upon normal keratinocytes were measured through a reduction in their survival at about 0.7 ± 0.08 for 100 µg/mL IONP_DOX_, and 0.56 ± 0.06 for 200 µg/mL IONP_DOX_.

**Figure 9:**
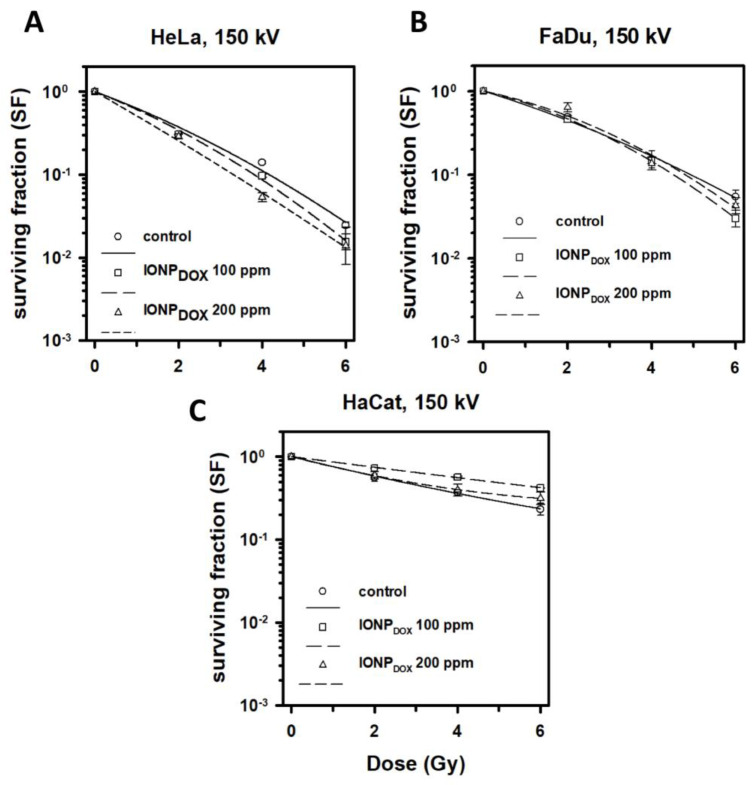
Clonogenic survival of 2D cultured cells exposed to 0, 100 and 200 μg/ mL IONP_DOX_ for 16 h, followed by 150 kV X-ray irradiation at different doses; results are shown as mean ± STDEV (n = 3): (**A**) HeLa cells, (**B**) FaDu cells, (**C**) HaCaT cells.

The irradiation of HeLa cells using 150 kV X-rays following the incubation with 100 μg/mL IONP_DOX_ led to a significant decrease in the clonogenic index at 4 Gy dose (*p* < 0.01, Figure 9A), with a dose modifying factor corresponding to a 0.1 survival fraction DMF_SF=0.1_= 1.29 ± 0.04. Similarly, the incubation with 200 μg/mL IONP_DOX_ induced a significant clonogenic inactivation at 4 Gy (*p* = 0.01, Figure 9A) with a DMF_SF=0.1_ = 1.55 ± 0.12. The two-way ANOVA test proved that there was a strong interaction between the analyzed factors, with a statistically significant difference at a 0.05 level between the populations by means of dose (F(3) = 259, *p* < 0.001), as well as by nanoparticle concentration (F(2) = 4, *p* < 0.05). The Fisher test confirmed that all data were statistically significant compared to the non-irradiated control; however, only the highest IONP_DOX_ concentration (200 μg/mL) determined a statistically significant modification of the cells’ survival rate, compared to the IONP-free group.

The incubation of squamous cell carcinoma FaDu cells with IONP_DOX_ did not induce any significant alteration in the cells survival rate following exposure at different doses of 150 kV X-rays, for none of the investigated concentrations (Figure 9B). These results suggest that the radiomodulating effect of the IONP_DOX_ was highly dependent on the cells type. Previously, it has been shown that the response to radiation alone [23,24], as well as to radioenhancers [25,26], is highly dependent on the cell type.

Moreover, the cell response to 150 kV irradiated human keratinocyte HaCaT demonstrated an increased resistance compared to tumor cells (Figure 9C). The HaCaT cells incubated with IONP_DOX_ for 16 h before irradiation showed a radio-modulating effect, proving that the IONP had a protective effect against ionizing radiation in normal human keratinocytes: 100 μg/mL IONP_DOX_ vs. control *p* < 0.001 at 4 Gy, and *p* < 0.05 for 6 Gy 200 μg/mL IONP_DOX_ vs. control NS. The main purpose of high atomic-number radioenhancers is to assure a high dose deposition at the tumor site, in order to increase the local cytotoxic response, while at the same time protecting the surrounding healthy tissues. Similarly, Bromma K. et al. [27] obtained a differentiated effect between normal and cancer cells, highlighted through differentiated cell uptake and DNA damage induction.

**Figure 10:**
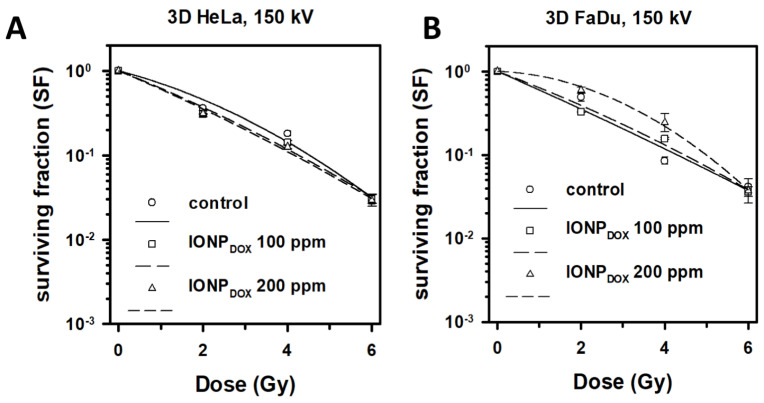
Clonogenic survival of 3D cultured cells exposed to 0, 100 and 200 μg/ mL IONP_DOX_ for 48 h, followed by 150 kV X-ray irradiation at different doses; results are shown as mean ± STDEV (n = 3): (**A**) HeLa cells, (**B**) FaDu cells.

The response of the tumor cells to IONPs and radiation treatment in 3D spheroids was different compared to the 2D cell models. The intrinsic cytotoxic effect on 3D tumor cell models differed depending on concentration and cell type. The effect of IONP_DOX_ in 3D FaDu spheroids induced a survival of 0.77 ± 0.23 at a concentration of 200 µg/mL, while the lower concentration did not induce a cytotoxic effect in the absence of ionizing radiation. In case of the 3D HeLa spheroids, the reduction in cell survival upon IONP_DOX_ incubation in absence of ionizing radiation was independent of the nanoparticle concentration, as follows: 0.68 ± 0.54 survival fraction in the case of 100 µg/mL, and 0.68 ± 0.55 for 200 µg/mL IONP_DOX_. In the case of the 3D HeLa cell model, a DMF_SF=0.1_ = 1.08 ± 0.1 for 100 µg/mL IONP_DOX_, and DMF_SF=0.1_ = 1.09 ± 0.1 for 200 µg/mL IONP_DOX_ in cells irradiated with 150 kV X-rays (Figure 10A). In the case of the 3D FaDu spheroids, the IONP had a rather protective effect against ionizing radiation (Figure 10B). Two-way ANOVA test showed that the results were significant by means of radiation dose variation, but not in the case of IONP concentration. The Fisher test confirmed these observations, and additionally showed that the incubation the HeLa spheroids with IONP previous to radiation treatment induced a radiosensitizing effect at the concentration of 200 μg/mL.

The radiomodulating effect in 3D tumor cell models was dependent on the nanoparticle penetration ability inside the spheroids [28,29], which was influenced by their morphology. Having a tight morphology, the squamous cell carcinoma model showed a radioprotective behavior following the nanoparticle exposure. The resistant behavior of this cell model may have been caused by the extensive hypoxic area, which was clearly emphasized in Figure 5D–F, and furthermore in Figure 7B. Although the nanoparticles were able to penetrate towards these areas, forming noticeable aggregate depositions in the hypoxic cells, the dual iron oxide-doxorubicin treatment was not sufficient to overcome the cells’ radioresistance. In the case of the cervical adenocarcinoma spheroids, the overall aspect of the cell model was diffuse, with loose morphology and few hypoxic areas (Figure 4 and Figure 6). Due to the large intercellular space, the IONPs migrated throughout the whole spheroid body (Figure 6B, intense blue areas in the cells).

The advantages of 3D cell cultures stand beyond the biomimetic architecture of the resulting culture [30]. Although it is known that cells in 3D environments exhibit a different phenotype as compared to 2D cell cultures [30,31], there are few studies that involved complex tumor models to prove the nanoparticles’ radiosensitizing behavior. For example, Motsumoto et al. [32] used Gd-capped SiO_2_ nanoparticles in order to obtain radiomodulating effects in tumor cell spheroids of human ovarian cancer. The results proved the nanoparticles’ activation ability using monochromatic X-rays at low energy (50.25 keV).

Approaching cancer treatment using multiple methods is frequently carried out in clinics. The present study was based on a combined effect of controlled chemotherapy as well as radiosensitization, using nanoparticle-based approaches. Other groups have used dual radiation treatment in order to obtain cytotoxic effects in nanoparticle-mediated radiotherapy in 3D cell models [33,34]. For example, Bulin et al. [33] used gold nanoparticles for radiosensitization in radiotherapy and photodymanic therapy at low doses. On the other hand, Rezaie [34] used magnetic nanoparticles covered with polymers to carry 5-iodo 2′deoxiuridine in 3D glioblastoma cell models, and to obtain radio-modulatory effects in dual therapy using 6MV X-ray and hyperthermia treatment. However, this is the first time that a dual nanoparticle-based radiosensitiziation and controlled drug delivery approach has been proven in 3D cell models.

## 3. Materials and Methods

### 3.1. Nanoparticle Synthesis and Characterization

Iron oxide nanoparticles were synthesized and encapsulated in polyethylene glycol (molecular weight 6 kDa) using a two-step method, starting from the co-precipitation synthesis of the crystalline cores, followed by the addition of a soft polymer shell in anhydrous conditions (1:1 polymer:nanoparticle ratio), as previously reported in Popescu et al. [16].

The resulting crystalline nanoparticle cores were composed of a single mineralogical phase identified as magnetite (Fe_3_O_4_), with a physical diameter of 12.82 ± 2.73 nm. Encapsulation in the polymer shells resulted in systems with a hydrodynamic diameter of 164.2 nm (polydispersity index (PDI) of 0.233 and zeta potential of 14.8 mV). The IONPs were able to encapsulate 1.11 wt% doxorubicin hydrochloride, which led to a mean hydrodynamic diameter of 369.1 nm (PDI 0.238 and zeta potential of −20.9 mV) [16].

### 3.2. Cell Culture

In order to assess the biological effect of the nanoparticle-based dual treatment, two tumor cell models were used: human cervical adenocarcinoma (HeLa) and human squamous cell carcinoma (FaDu), as well as a normal cell model: human keratinocytes (HaCaT) (ATCC, Manassas, VA, USA). The cells were cultured in DMEM, supplemented with 10% FBS and 0.1% penicillin–streptomycin (HeLa and HaCaT), and in MEM, supplemented with 10%FBS, 0.1% pyruvate and 0.1% penicillin–streptomycin (FaDu), in standard conditions of temperature and humidity (37 ± 2 °C, 5 ± 1% CO_2_, more than 90% humidity). The 2D cell cultures were prepared by seeding the cells in normal flat and adherent tissue culture plates (TPP, Trasadingen, Schaffhausen, Switzerland) at different concentrations, as described below in Section 3.3.

#### 3D Cell Model

The 3D cell models were obtained using the liquid-overlay technique [18]. For this, different cell concentrations of each type (HeLa and FaDu—between 250–20,000 cells) were seeded in U-shaped 96-well plates with non-adherent properties (Corning, Sigma Aldrich, St. Louis, MO, USA). The cells were incubated for 72 h in standard conditions of temperature and humidity, in order to allow assembly of the spheroids. The cell culture medium suitable for each cell line was refreshed every 3 days, and bright-field images of the spheroids were acquired using a Nikon optical microscope (Minato, Tokyo, Japan) equipped with an Olympus Pen Lite E-PLS 144 camera (Shinjuku, Tokyo, Japan), with a 4× objective. The spheroid measurements were conducted using ImageJ software (National Institute of Health, Bethesda, MA, USA).

The characterization of the tumor spheroids was carried out at 72 h following the seeding (first day of evaluation), and in the last day of evaluation (day 7 for HeLa cells, and day 14 for FaDu).

The spheroids were incubated with 30 µM bromodeoxyuridine (BrdU) and 300 µM pimonidazole (Pimo), 2 h before fixing. Following the incubation, the spheroids were collected and fixed overnight using Roti-Histofix 4% (Carl Roth, Karlsruhe, Germany). The cells were then washed with ethanol of increasing concentrations (50%, followed by 70%) for 15 min, and transferred into plastic molds to be embedded in Histogel (Thermo Fisher Scientific, Waltham, MA, USA). After solidification, the blocks were transferred in histo-cassettes, and routine overnight desiccation procedures were applied.

The paraffin embedding was carried out using TissueTEC (Sakura Finetek, Alphen aan den Rjin, The Netherlands) equipment, and the resulting blocks were frozen at −20 °C for 2 h before sectioning. Thus, 4-micrometer sections were obtained using a HM 355S Microtome (Thermo Fisher Scientific, Waltham, MA, USA), and collected on microscopy slides. The dewaxing procedure was carried out via successive immersion of the samples in xylol and ethanol solutions with decreasing concentrations (100–40%).

For immunohistochemical staining, the samples were prepared through antigen unmasking using a citrate solution. Following this, a blocking procedure using a solution of goat serum was carried out to precede the pimonidazole staining, and a blocking procedure using a solution of rabbit serum was carried out, to precede the Bromodexyuridine staining. The primary antibody reaction was conducted overnight at 4 °C, using a Hypoxyprobe-1 Omni kit PAb 2627(AP) Rabbit antisera (Hypoxyprobe, Burlington, MA, USA) for Pimo, and ab6326 Rat mAb to BrdU (Abcam, Cambridge, UK) for BrdU, according to the producers’ specifications. Afterwards, the unreacted antibody was removed, and the secondary antibody was added to bind for 1 h at room temperature. All of the cells were colored using hematoxylin (violet), and antibody-marked cells were colored using a peroxidase system (brown).

In order to choose a suitable tumor model for the iron oxide nanoparticles evaluation, different initial cell concentrations were used to form the spheroids through the liquid-overlay technique, followed by incubation in standard conditions of temperature and humidity (37 °C, 5 ± 1% CO_2_, more than 90% humidity). The median diameter of the spheroids, as well as their morphology, were monitored for 2 weeks. While squamous cell carcinoma showed a dense and tight morphology inside the resulting spheroids, the cervical adenocarcinoma cell spheroids were characterized by a loose morphology, with the cell density decreasing towards the center of the spheroid. Models with an initial median diameter of 0.5 ± 0.1 mm were obtained (Supplementary Materials, Section S1, Figures S1–S3).

### 3.3. Nanoparticle Treatment and Internalization

For the 2D cell culture, 100,000 cells/well were seeded in 12-well plates and incubated for 4 h. After this time, the culture medium was replaced with fresh medium containing 100 and 200 µg/mL nanoparticles. Cells were incubated for another 16 h, then washed 3 times with PBS and detached and centrifuged (1700 rpm, 5 min), in order to completely remove the non-internalized nanoparticles. Afterwards, the supernatant was replaced with culture medium without nanoparticles, and the cells were seeded again on 10 mm coverslips at a density of 25,000 cells/slide and incubated for another 24 h. Following this step, cells were fixed with 3.7% paraformaldehyde in PBS for 10 min. Nuclei marking was carried out with hematoxylin, and nanoparticles were colored with potassium hexacyanoferrate trihydrate (Prussian Blue, Merck-Sigma Aldrich, Darmstadt, Germany).

HeLa and FaDu spheroids were obtained and treated with iron oxide nanoparticles at concentrations of 100 and 200 µg/mL. After 0, 4, 16, 24, 48 and 72 h of incubation in the presence of the nanoparticles, the spheroids were collected, washed 3 times with PBS, and fixed overnight in Roti-Histofix 4%. The spheroid preparation for immunohistochemical analysis was carried out as described in Section 3.2. 3D cell model. Transversal sections of 4 µm in thickness were obtained from the paraffin blocks containing the embedded spheroids. Following dewaxing, the samples were incubated in the presence of 1:1 potassium hexacyanoferrate trihydrate 2%:HCl 1 M for 15 min at 37 °C, for Prussian Blue staining of iron oxide nanoparticles. The nuclei coloring was carried out with Hoechst, and the coloring of the cells with hematoxylin. The images were acquired using an Axio Observer microscope (Zeiss, Jena, Germany).

### 3.4. In Vitro Irradiation and Clonogenic Survival Assay

Cell samples for the clonogenic evaluation of iron oxide nanoparticles radiosensitization were prepared as described in Section 3.3.

2D cell cultures were obtained by seeding 100,000 cells/well in 12-well plates, and incubating them at standard conditions of temperature and humidity for 4 h, in order to allow for their attachment. Then, the culture medium was replaced with fresh culture medium containing nanoparticles (at concentrations of 100 and 200 µg/mL), and the cells were incubated for another 16 h. After this time, the cells were carefully washed 3 times with PBS, and fresh medium with no nanoparticles was added.

3D cell cultures were obtained by seeding 20,000 cells/well for FaDu and 1000 cells/well for HeLa, in low-attachment round-shaped 96-well plates and incubated for 3 days, in order to form the spheroids. Afterwards, nanoparticles were added at concentrations of 100 and 200 µg/mL, and incubated for another 48 h. After this time, the spheroids were washed 3 times with PBS, and fresh medium with no nanoparticles was added to each well.

The as-prepared samples were irradiated using medium-energy (150 kV) X-rays, at doses between 0–6 Gy at a penetration depth of 2 mm (Gulmay D3300 X-ray device (Gulmay Ltd., Chertsey, UK), operating at 150 kV at a dose rate of ~1.9 Gy/min with a 2.4 mm Al filter. Following the radiation treatment, the cells were detached in single-cell suspension using 1% trypsin in PBS or Accutase, and seeded at a concentration of 200–500 cells/well in 6-well plates. The cells were incubated for 14 days in standard conditions of temperature and humidity. Following this time, the cells were fixed using a solution of acetic acid/methanol, and colored using crystal violet, according to [17,35]. Counting and interpretation was conducted as described in [17,35,36], as follows: colonies counting more than 50 cells were scored, and the surviving fraction (SF) was calculated as the ratio between the resulting number of colonies and the seeded cell numbers in both (un)irradiated samples [35,36]; in order to represent the surviving fraction (SF), a linear-quadratic fit (ln(SF) = −(αD + βD^2^), where D is the dose, α is the linear component of cell survival and β is the quadratic component of the cell survival) was applied using the non-linear regression tool in SigmaPlot 15 (Systat Software GmbH, Erkrath, Germany) [35,36]; to evaluate the radiosensitizing potential of the IONP_DOX_, normalization to the control was applied (IONP_DOX_, non-irradiated cells) [17,36].

### 3.5. Statistical Analysis

All of the experiments were carried out in triplicate. Results were shown as ±STDEV, and the statistical analysis was performed using two-way ANOVA (Fisher) and Student’s *t* test, where * *p* < 0.05, ** *p* ≤ 0.01 and respectively *** *p* ≤ 0.001.

## 4. Conclusions

The radiosensitization potential of the proposed iron oxide-based nanosystems for doxorubicin delivery was dependent on the nanoparticle concentration, but also on the dose of ionizing radiation. Moreover, there was a clear differentiated effect between normal and tumor cells, evidenced as a radioprotective effect in the case of the HaCaT cells, and as a radiosensitizing effect for HeLa (DMF_SF=0.1_ = 1.29 ± 0.04 for 100 μg/mL IONP_DOX_, DMF_SF=0.1_ = 1.55 ± 0.12 for 200 μg/mL IONP_DOX_, *p* < 0.05) and FaDu (NS), although IONPs were similarly internalized in all models through a peri-nuclear pattern. The arrangement of the cells in culture was also a factor that influenced the radiotherapy response. The morphology of the 3D spheroids was decisive for the nanoparticle uptake and trafficking: in case of human cervical adenocarcinoma models, the nanoparticles mostly interacted with proliferative cells, spread through the whole volume of the spheroid; meanwhile, in the case of squamous cell carcinoma models, the IONPs penetrated the spheroid and interacted with both proliferative and hypoxic cells. The loose morphology of the HeLa 3D model determined a radiosensitizing effect of IONP (DMF_SF=0.1_ = 1.08 ± 0.1 for 100 µg/mL IONP_DOX_ NS, and DMF_SF=0.1_ = 1.09 ± 0.1 for 200 µg/mL IONP_DOX_, *p*< 0.05), but this was not evidenced in the case of the FaDu 3D cell model. These results substantiate the multimodal treatment ability of polyethylene-glycol-iron-oxide nanosystems for doxorubicin delivery and radiosensitization of 3D tumor cell models in vitro.

## Data Availability

Not applicable.

## Supplementary Materials

The following supporting information can be downloaded at: https://www.mdpi.com/article/10.3390/ijms24032198/s1, Figure S1. FaDu spheroids morphology during the first day of monitorization; Figure S2. HeLa spheroids morphology during the first day of monitorization; Figure S3. Growth curve at different initial cell concentrations; Figure S4. Immunohistochemical characterization of FaDu spheroids using brightfield microscopy; Figure S5. Immunohistochemical characterization of HeLa spheroids using brightfield microscopy; Figure S6. Brightfield microscopy images of sections at different depths in FaDu spheroids, previously incubated with IONP_DOX_; Figure S7. Brightfield microscopy images of sections at different depths in FaDu spheroids, previously incubated with IONP_DOX_; Figure S8. Brightfield microscopy images of sections at different depths in HeLa spheroids, previously incubated with IONP_DOX_; Figure S9. Brightfield microscopy images of sections at different depths in HeLa spheroids, previously incubated with IONP_DOX_;
