# Supplementary material for: Nanoparticle-Mediated Drug Delivery of Doxorubicin Induces a Differentiated Clonogenic Inactivation in 3D Tumor Spheroids In Vitro
Source: Int J Mol Sci. 2023 Jan 22;24(3):2198. doi: 10.3390/ijms24032198 (PMC9916819; doi:10.3390/ijms24032198)
Supplement: Supplementary file 1 [file ijms-24-02198-s001.zip › ijms-2158896-supplementary.pdf]

Supplementary Material of the article

Nanoparticle-Mediated Drug Delivery of Doxorubicin Induces a Differentiated Clonogenic Inactivation in 3D Tumor Spheroids In Vitro

Roxana Cristina Popescu [1,2,3], Verena Kopatz [3], Ecaterina Andronescu [2**], Diana Iulia Savu[1],* and Wolfgang Doerr [3†]

[1] *Department of Life and Environmental Physics, National Institute for R&D in Physics and Nuclear Engineering "Horia Hulubei", Reactorului street, 30, 077125, Magurele, Romania*
[2] *Department of Bioengineering and Biotechnology, Department of Science and Oxide Materials and Nano-materials, Politehnica University of Bucharest, Splaiul Independentei, 313, 060042, Bucharest, Romania*
[3] *Department of Radiation Oncology, Medical University of Vienna, Waehringer Guertel, 18-20, 1090, Vienna, Austria*
[†] *Deceased*
* *Correspondence: dsavu@nipne.ro, ecaterina.andronescu@upb.ro*

Supplementary Material Section S1. Obtaining and characterization of tumor spheroids.

The 3D cell models were obtained using the liquid-overlay technique. For this, different cell concentrations of each type (HeLa and respectively FaDu- between 250- 20000 cells) were seeded in U-shaped 96 well plates with non-adherent properties (Corning, Sigma Aldrich). The cells were incubated for 72h in standard conditions of temperature and humidity, to allow the assembling of the spheroids. The cell culture medium suitable for each cell line was refreshed every 3 days and brightfield images of the spheroids were acquisitioned using a Nikon optical microscope (Minato, Tokyo, Japan), equipped with an Olympus Pen Lite E-PLS 144 camera (Shinjuku, Tokyo, Japan) with a 4x objective. The spheroid measurements were done using the ImageJ software (National Institute of Health, Bethesda, MA, USA).
The characterization of the tumor spheroids was done at 72h following the seeding (first day of evaluation- Figures S1, S2), respectively in the last day of evaluation (day 7 for HeLa cells, respectively day 14 for FaDu).

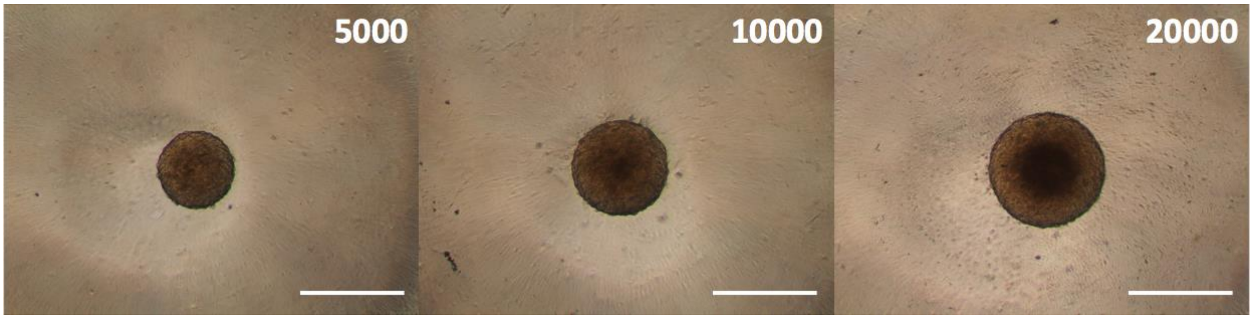

Figure S1. FaDu spheroids morphology during the first day of monitoring, at different initial cell seeding concentrations; measure bar is 0.5 mm.

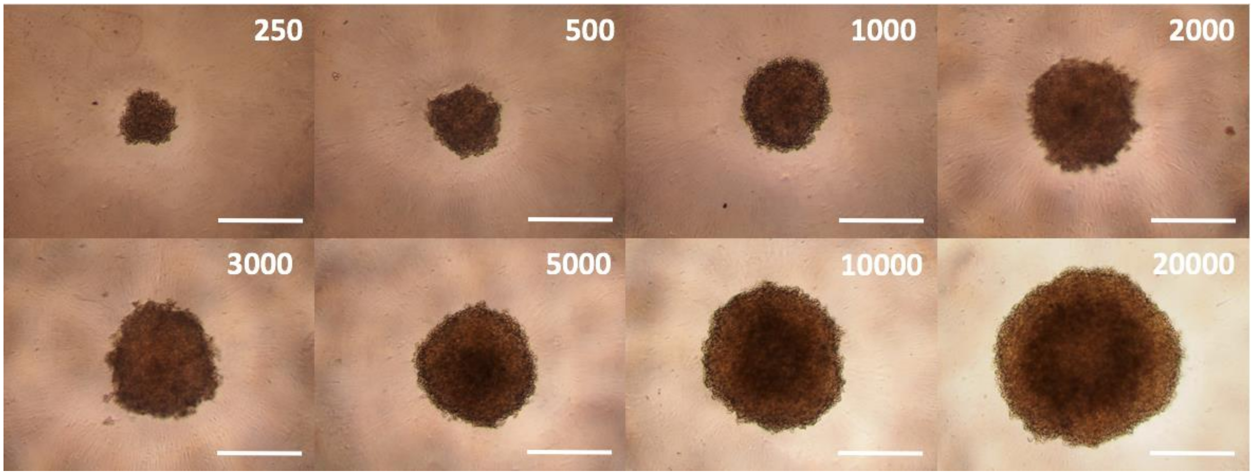

Figure S2. HeLa spheroids morphology during the first day of monitorization, at different initial cell concentrations; measure bar is 0.5 mm.

The spheroids were incubated with 30 µM Bromodeoxyuridine (BrdU) and 300 µM Pimonidazole (Pimo), 2h before fixing. Following the incubation, the spheroids were collected and fixed overnight using Roti-Histofix 4% (Carl Roth, Karlsruhe, Germany). The cells were then washed with ethanol of increasing concentrations (50%, followed by 70%) for 15 minutes and transferred into plastic molds to be embedded in Histogel (Thermo Fisher Scientific, Waltham, MA, USA). After solidification, the blocks were transferred in histo-cassettes and overnight desiccation procedures were applied.

The paraffin embedding was done using a Tissue Tek TEC (Sakura Finetek, Alphen aan den Rjin, Netherlands) equipment and the resulting blocks were frozen at -20°C, 2h before sectioning. Thus, 4µm sections were obtained using a HM 355S Microtome (Thermo Fisher Scientific, Waltham, MA, USA) and collected on microscopy slides. The dewaxing procedure was done by successive immersion of the samples in Xylol and ethanol solutions with decreasing concentrations (100%-40%).

For the immunohistochemical staining, the samples were prepared through antigen unmasking using a citrate solution. Following this, the blocking procedure using a solution of goat serum was done, to precede the Pimonidazole staining, respectively the blocking procedure using a solution of rabbit serum was done, to precede the Bromodexyuridine staining. The primary antibody reaction was done overnight, at 4°C using Hypoxyprobe-1 Omni kit PAb 2627(AP) Rabbit antisera (Hypoxyprobe, Burling-ton, MA, USA) for Pimo and respectively ab6326 Rat mAb to BrdU (Abcam, Cam-bridge, UK) for BrdU, according to the producers' specifications. Afterwards, the un-reacted antibody was removed, and the secondary antibody was added to bind during 1h, at room temperature. All cells were colored using hematoxyline (violet) and anti-body-marked cells were colored using a peroxidase system (brown).

In order to choose a suitable tumor model for the iron oxide nanoparticles evaluation, different initial cell concentrations were used to form the spheroids through the liquid overlay technique, following incubation in standard conditions of temperature and humidity (37°C, 5±1% CO_2, more than 90% humidity). The medium diameter of the spheroids, as well as their morphology, were monitored for 2 weeks. While squamous cell carcinoma proved a dense and tight morphology

inside the resulted spheroids, the cervical adenocarcinoma cell spheroids were characterized by a loose morphology, the cell density decreasing towards the center of the spheroid. Models with an initial medium diameter of 0.5±0.1 mm were obtained (Figure S3).
The number of cells was calculated using the Analyze tools in the ImageJ software (National Institute of Health, MA, USA). The images for each category were loaded in the program and thresholds were applied to count the Brdu/ Pimo- positive cells. The percentage of Brdu/ Pimo-positive cells was calculated as related to the total number of cells in each spheroid slice. The necrotic area was calculated as percentage of the spheroid slice area. At least 10 samples were measured for each category. Results were presented as mean ± SEM.
FaDu cells were seeded at concentrations of 20000, 10000, 5000 cells/ spheroid, resulting tumor models with round shape and initial diameters of 0.57±0.03, 0.45±0.01, 0.37±0.02 (Figure S3- A). The resulting spheroids monitoring was possible until the 14th day, the mean diameter measurements at this time interval being 0.47±0.05, 0.4±0.04, 0.37±0.06 (Figure S3- A). While spheroids from the 5000 cells/spheroid initial cell concentration revealed a small diameter increase during the monitoring time, the spheroids in the 10000 cells/spheroid initial cell concentration did not show any alteration of their dimension, while the spheroids in 20000 cells/spheroid initial cell concentration showed a decrease of their dimension proportional with the incubation time (Figure S3- A). By analyzing the growth curves (Figure S3- A), one can observe that FaDu spheroids initially go through a process of compacting before they start to grow again. By taking all these facts into account, the initial concentration of 20000 cells/spheroid was chosen to obtain the squamous cell carcinoma 3D model.
In case of HeLa model, the high cell concentration spheroids proved an accelerated growth, with diameters of 1.59±0.05, 1.27±0.06, 1.11±0.04, 0.95±0.05, 0.87±0.053 mm for 20000, 10000, 5000, 3000, respectively 2000 cells/spheroid concentration at 3 days following seeding (Figure S3- B). The spheroids monitoring was not possible after this time interval due to excessive growing. A second experimental group was done for reduced initial cell concentrations of 250, 500, respectively 1000 cells, which showed initial diameters of 0.4±0.04, 0.48±0.05, respectively 0.6±0.05 mm (Figure S3- C). The monitoring of these spheroids' morphology was possible up to the 7th day of incubation, mainly due to excessive diameter growth (Figure S3- C). By taking these observations into consideration, an initial concentration of 1000 cells/spheroid was selected to obtain the cervical adenocarcinoma 3D model.

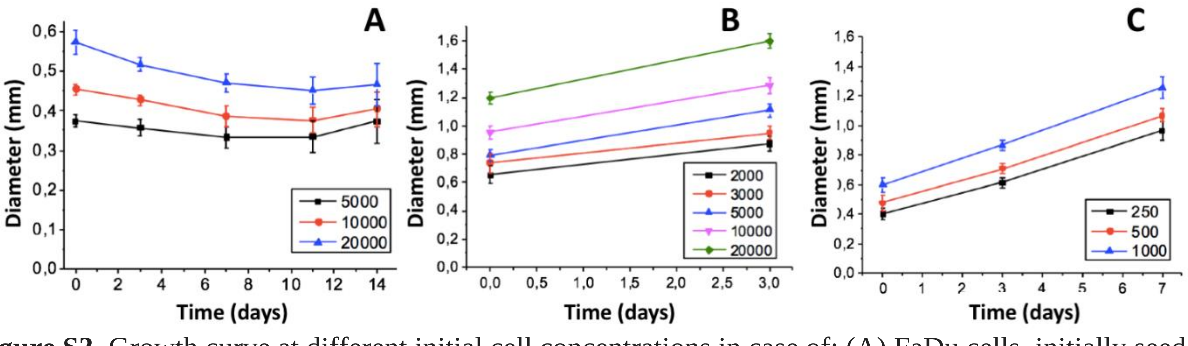

Figure S3. Growth curve at different initial cell concentrations in case of: (A) FaDu cells, initially seeded at 5000, 10000, respectively 20000 cells/ spheroid; (B) HeLa cells, initially seeded at 2000, 3000, 5000, 10000, respectively 20000 cells/ spheroid; (C) HeLa cells, initially seeded at 250, 500, respectively 1000 cells/ spheroid;

The immunohistochemical analysis of the transversal sections of the tumor spheroids was done in order to identify the proliferative/ hypoxic/ necrotic regions. In case of squamous cell carcinoma model, the BrdU marking indicated an increased proliferation rate of the cells in the peripheral areas of the spheroid, with few BrdU-positive cells towards the center of the spheroid (Figure S4- A, C). These observations were valid for the investigations done in the initial day (Figure S4- A), as well as in the 14th day of monitoring (Figure S4- C). In the first day of investigation, a well-defined necrotic area was observed in the center of the spheroid, surrounded by an area positive for Pimo proving the central hypoxia of the cells (Figure S4- B). In Figure S4- B, D the gradient coloration shows the oxygen inability to penetrate the spheroid, which increases towards the spheroid center. The necrotic area was not observed for samples investigated at 2 weeks of incubation and growth, probably as a result of gradual spheroid contraction in the FaDu model (Figure S4- D). Moreover, these samples did not show any evidence of hypoxic areas (Figure S4- D). In case of Fadu spheroids, a total number of 18.35±2.54% Brdu-positive cells were counted, respectively a total number of 46.68±1.33% number of hypoxic cells. The area of necrotic cells was well defined and about 8.16±1.61% in each slice.

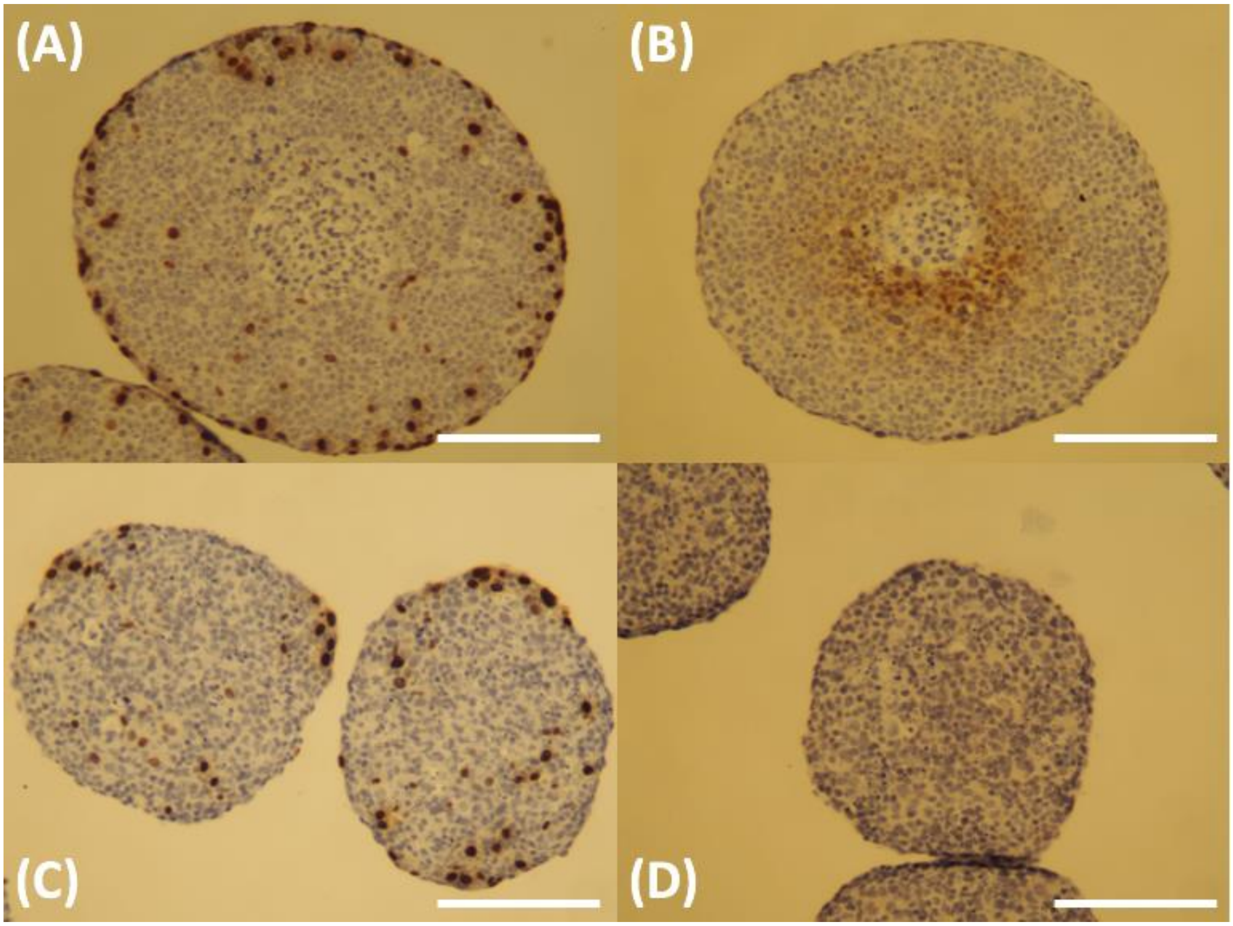

Figure S4. Immunohistochemical characterization of FaDu spheroids (20000 cells/spheroid) using brightfield microscopy: (A, C) bromodeoxyuridine staining of proliferative cells, represented with dark brown; (B, D) pimonidazole staining of hypoxic cells, represented with light blue; hematoxylin staining of FaDu cells; measure bar is 0.2 mm; (A-B) 3 days following seeding and (C-D) end of monitorization time (14 days); measure bar is 100 µm.

Regarding the HeLa spheroids, in the first day of monitoring, transversal sections confirmed their loose morphology, which can easily disintegrate due to manipulation (Figure S5- A, B). Specific marking of proliferative areas showed a homogenous dispersion of BrdU- positive cells while no hypoxic areas could be identified (Figure S5- A). At 14 days of incubation, cervical adenocarcinoma spheroids showed a round morphology with loose structure (Figure S5- C, D). A secondary proliferative spheroid was identified inside the initial spheroid, which is most probably formed to take place of the necrotic area (Figure S5- C, D). Due to the rather loose architecture of the spheroid matrix, the oxygen and nutrients can easily penetrate towards the central area, determining a continuous growth. Both hypoxic and proliferative cells were identified to be dispersed in the spheroids (Figure S5- B, D). In case of HeLa spheroids, a total number of 46.91±5.25% Brdu-positive cells were counted, respectively a total number of 42.93±4.93% Pimo-positive cells. The necrotic core was not established, as necrotic areas were spread around the whole spheroid. Due to the loose morphology of the cervical adenocarcinoma spheroids with large in-between spaces, it was not possible to identify these areas.

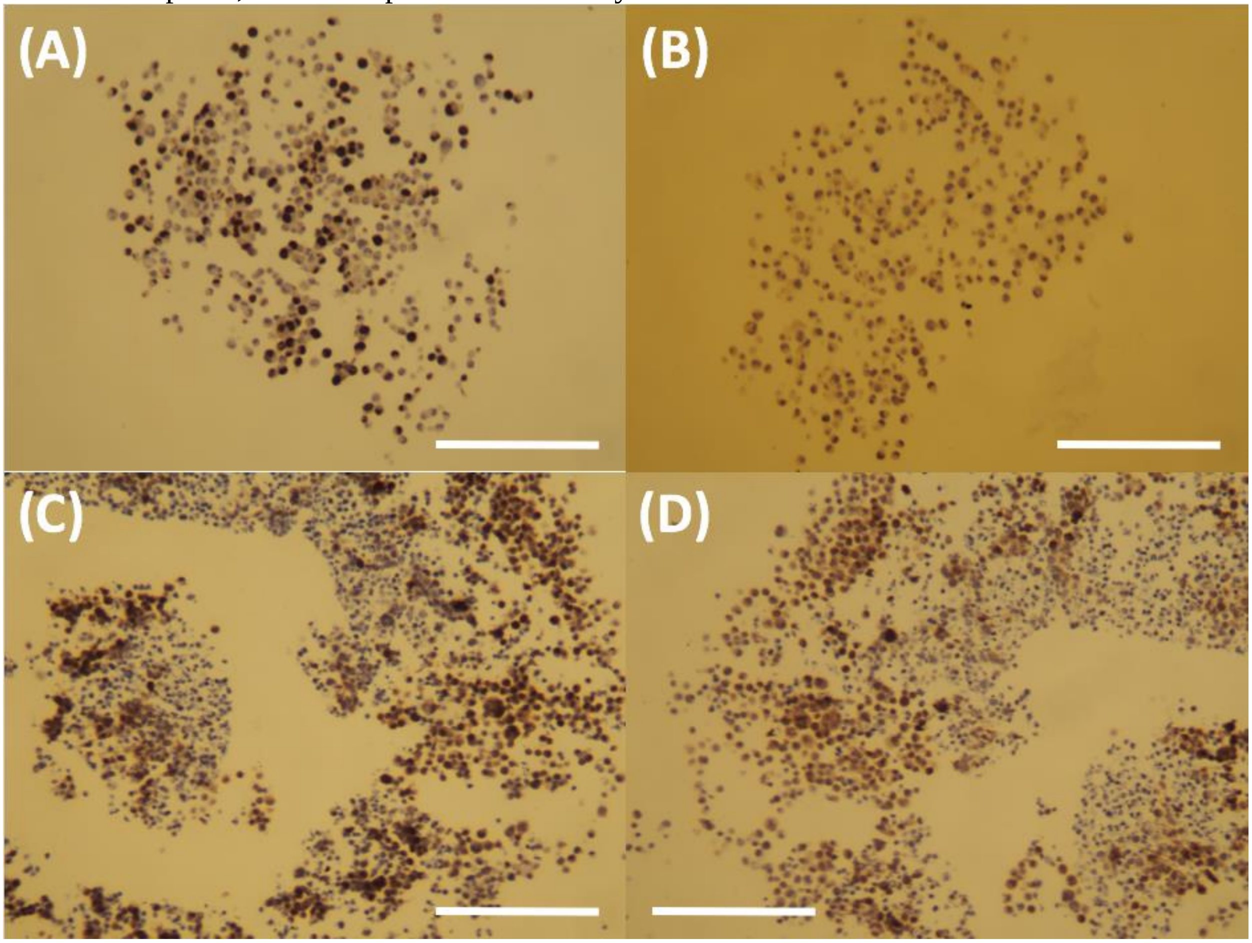

Figure S5. Immunohistochemical characterization of HeLa spheroids (1000 cells/ speroid) using brightfield microscopy: (A, C) bromodeoxyuridine staining of proliferative cells, represented with dark brown; (B, D) pimonidazole staining of hypoxic cells, represented with light blue; hematoxylin staining of FaDu cells; measure bar is 0.2 mm; (A-B) 3 days following seeding and (C-D) end of monitorization time (7 days); measure bar is 100 μm.

Supplementary Material Section S2. IONP internalization in tumor spheroids.

HeLa and respectively FaDu spheroids were obtained and treated with iron oxide nanoparticles at concentrations of 100, respectively 200 µg/mL. After 0, 4, 16, 24, 48, respectively 72h incubation in the presence of nanoparticles, the spheroids were collected, washed for 3 times with PBS and fixed overnight in Roti-Histofix 4%. The spheroid preparation for immunohistochemical analysis was done as described in section 3.2. *3D cell model*. Transversal sections of 4 µm thickness were obtained from the paraffin blocks containing the embedded spheroids. Following dewaxing, samples were incubated in presence of 1:1 potassium hexacyanoferrate trihydrate 2% : HCl 1M, during 15 minutes, at 37°C, for Prussian Blue staining of iron oxide nanoparticles. The nuclei coloring was done with Hoechst, respectively the coloring of the cells with hematoxylin. The images were acquired using an Axio Observer microscope (Zeiss, Jena, Germany) (Figures S6-S9).

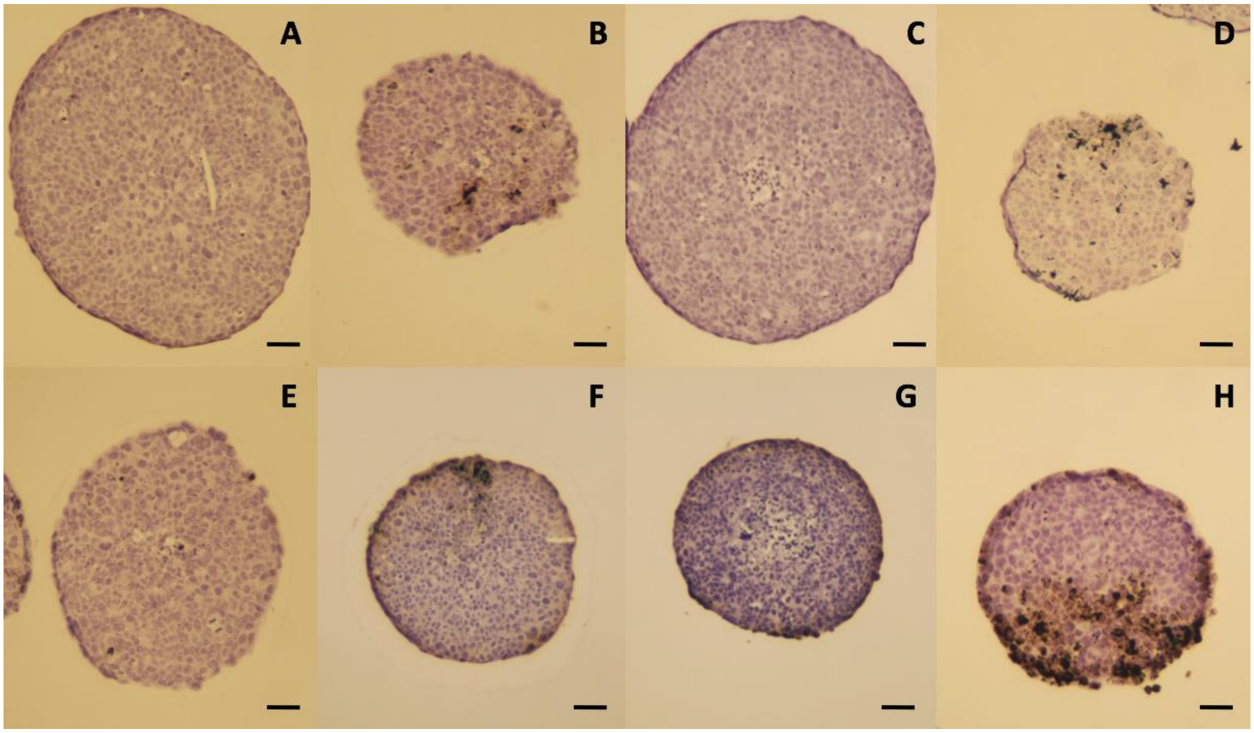

Figure S6. Brightfield microscopy images of sections at different depths in FaDu spheroids, previously incubated with $IONP_{DOX}$ at a concentration of 100 µg/mL for different time intervals: (A-B) 16h, (C-D) 24h, (E-F) 48h, (G-H) 72h; violet- hematoxylin, brown – $IONP_{DOX}$; measure bar is 20 µm;

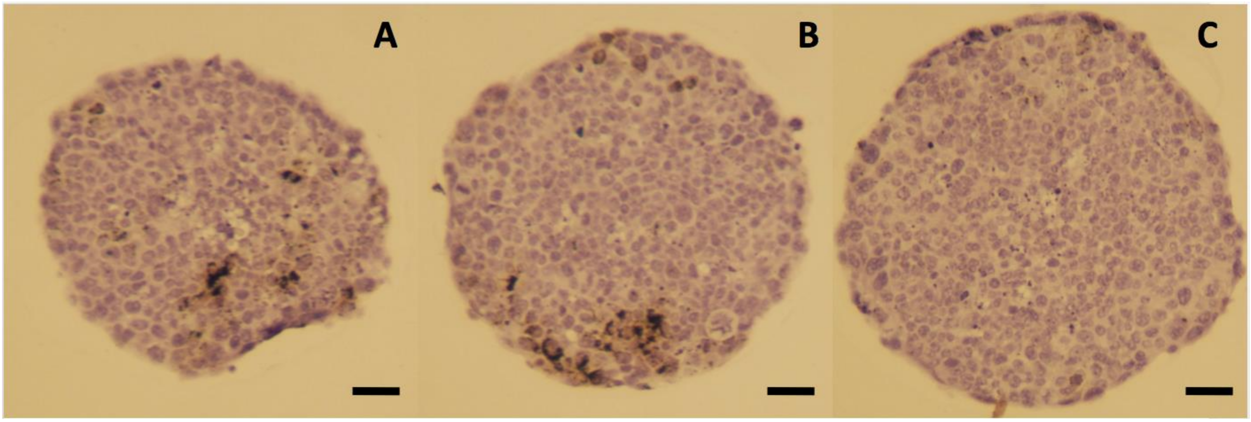

Figure S7. Brightfield microscopy images of sections at different depths in FaDu spheroids, previously incubated with $IONP_{DOX}$ at a concentration of 100 µg/mL for 48h; violet- hematoxylin, brown – $IONP_{DOX}$; measure bar is 20 µm;

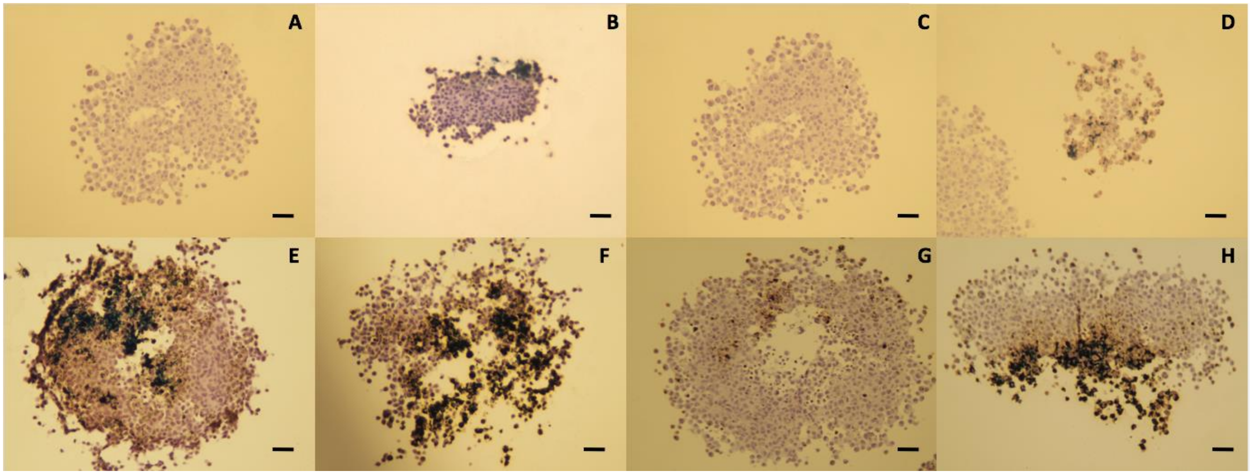

Figure S8. Brightfield microscopy images of sections at different depths in HeLa spheroids, previously incubated with $IONP_{DOX}$ at a concentration of 100 µg/mL for different time intervals: (A-B) 16h, (C-D) 24h, (E-F) 48h, (G-H) 72h; violet- hematoxylin, brown – $IONP_{DOX}$; measure bar is 20 µm;

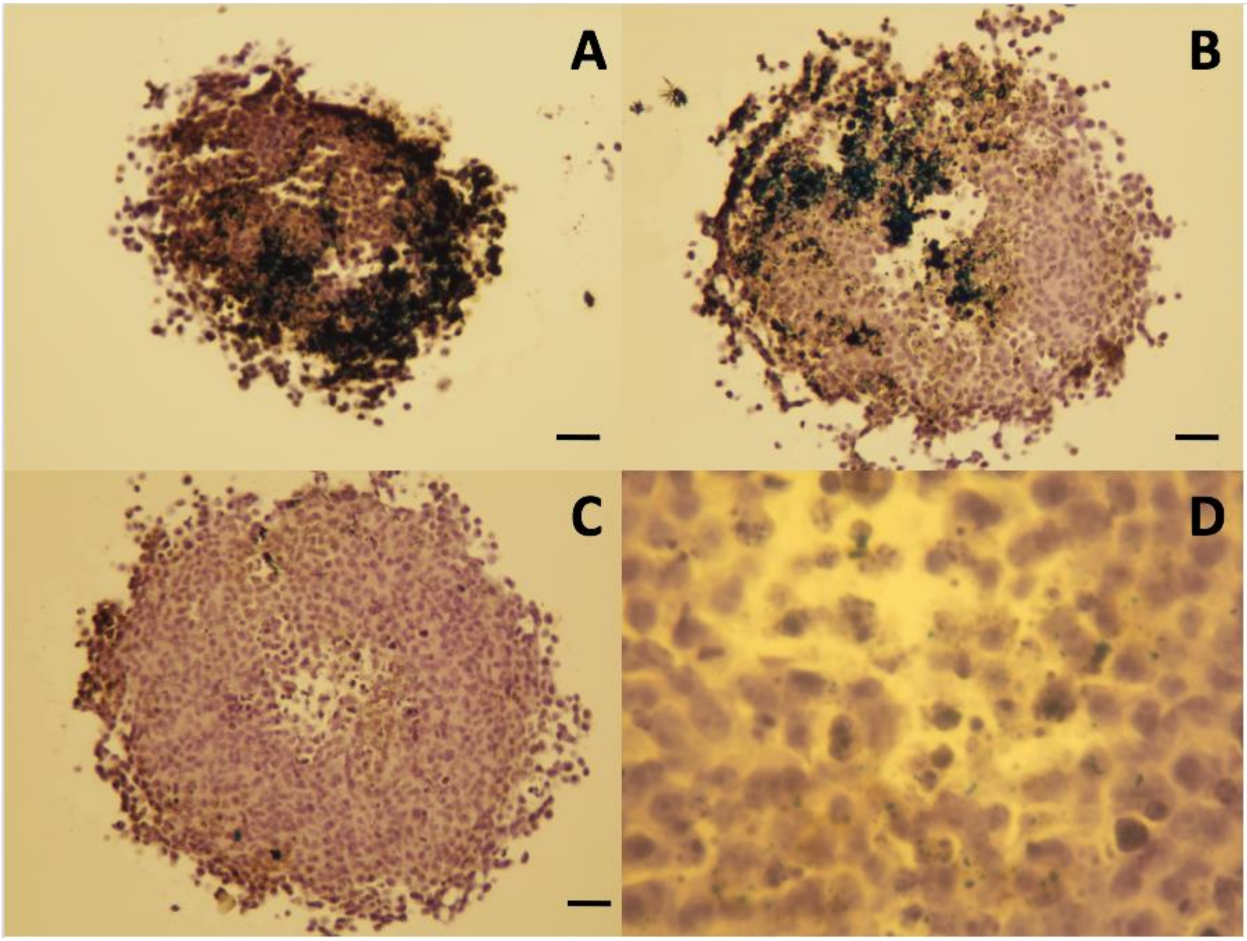

Figure S9. Brightfield microscopy images of sections at different depths in HeLa spheroids, previously incubated with $IONP_{DOX}$ at a concentration of 100 µg/mL for 48h; violet- hematoxylin, brown – $IONP_{DOX}$; measure bar is 20 µm;
